# School Environments and Elementary School Children’s Well-Being in Northwestern Mexico

**DOI:** 10.3389/fpsyg.2020.00510

**Published:** 2020-03-19

**Authors:** César Tapia-Fonllem, Blanca Fraijo-Sing, Victor Corral-Verdugo, Glenda Garza-Terán, Melanie Moreno-Barahona

**Affiliations:** ^1^Programs of Master and Doctorate in Psychology, University of Sonora, Hermosillo, Mexico; ^2^Programs of Master and Doctorate in Social Sciences, University of Sonora, Hermosillo, Mexico

**Keywords:** school environment, well-being, positive school, children, elementary school

## Abstract

School environment refers to the set of relationships that occur among members of a school community that are determined by structural, personal, and functional factors of the educational institution, which provide distinctiveness to schools. The school environment is an important factor when evaluating student well-being. Previous findings have shown that variables such as physical, academic, and social dimensions influence school environments. This research seeks to explain the relationship between school environment and the well-being of primary education students. To carry out this research, a total of 405 students from four public elementary schools in northwestern Mexico were selected to participate. The instrument used to measure the variables and the relationship of school environment and well-being is based on the three dimensions of school environment proposed by Kutsyuruba et al. (2015): Physical, social, and academic. Statistical analyses were carried out to determine the reliability and validity of the measurement scales using SPSS V20 and EQS software. Confirmatory factor analysis models were tested to determine the construct validity of each scale; then, an analysis via structural equation modeling was made to form an explanatory model obtaining acceptable practical and statistical indicators. Among the relationships in this study, our research identified the variable of school environments as an outcome determined by physical, academic, and social factors. School environment and student well-being variables were also found to be correlated.

## Introduction

The study of the physical, social, and academic (curricular) conditions of the environment and the administrative organization of schools have been related to school environments and the well-being of students (Corral-Verdugo et al., 2015). Nowadays, it has become more common to find empirical studies that identify the impact of school environments on student well-being. For example, safe school environments and student well-being have been found to be significantly and strongly interrelated variables on research of various kinds of students’ needs (Kutsyuruba et al., 2015).

Primarily, research of positive school environment is focused on physical conditions: density, privacy, activity areas, open spaces, and, even, green areas. Some of the most researched effects from physical elements have been the ones resulting from noise, lighting and colors, temperature and humidity, decoration, and furniture, since they contain properties that have effects on people’s behavior; nevertheless, despite having found evidence of these effects, the results are not considered entirely conclusive (Olivos and Amérigo, 2010). The quality of these conditions in school infrastructure can have direct effects on the behavior and cognitive, social, and emotional development of children (Prescott and David, 1976; Wohlwill and Heft, 1987; Moore et al., 2003). In other words, the school space is considered a didactic agent that helps to offer optimal physical conditions for the development of the teaching-learning process. Likewise, it allows for the creation of an adequate environment for the development of students’ abilities, fostering their autonomy as well as teacher motivation.

Romañá (1994) focused on the role that the environment takes as an object of attention for learning. There are three ideas about how it has been addressed: (a) conceiving the environment as an educator: the nature of physical elements of the environment as socializing agents themselves; (b) considering it as an educational object for the valuation and conservation of the environment, and (c) and conceiving it as an educational or didactic resource; in other words, as a pedagogical utility factor.

Olivos and Amérigo (2010) performed a historical review and background check on the study of the connection between environment and education and identified that it had been studied in the fields of pedagogy, where it had been called “environmental pedagogy” (Göttler, 1955) or “mesological pedagogy” (Zaniewski, 1952); and psychology, under the term “classroom ecology” (Sommer, 1967; Weinstein, 1979). Other authors have also underlined how the emotional dimension is an important component in the development of evaluation competences, such as for example, the aesthetic evaluation experience, and we argue that this component could also be relevant for the evaluation of school environments (e.g., Mastandrea, 2014; Mastandrea and Crano, 2019).

At the end of the 20th century, environmental psychology focused its attention on the study of school environments, specifically on aspects of practical conditions such as ergonomics and architecture, considering particular physical aspects of the school environment and its role in the process of teaching learning and even associating it with academic performance (Holahan, 1986; Gump, 1987; Bell et al., 1990; Gifford, 2007; Amedeo et al., 2008).

However, there are always challenges for the design and management of educational spaces and they overcome the traditional difficulties of improving the teaching-learning process in conflictful conditions resulting from social interaction within school environments. A wide range of studies has found a reduction of negative or violent behaviors that are usually present in schools are due to management changes in physical environments (Bosworth et al., 2011; Steffgen et al., 2013; Cornell et al., 2015). Current trends in educational intervention consider the promotion of positive personal interactions as a priority and as a cause or consequence of harmonious activities of the school with its environment, putting integration into practice (Corral-Verdugo et al., 2015).

It is in the second decade of the 21st century when special attention was paid to the study of school environments (Bernardes and Vergara, 2017), school climate (Wang and Degol, 2016; Maxwell et al., 2017) and its connection with student well-being (Bird and Markle, 2012; Borkar, 2016).

Currently, research on physical aspects in school environments has gained attention as a result of the theoretical relevance of the human-environment link, the new conceptions about the importance of social interactions in the educational environment, and questions about the objectives of education in the modern world (Aldridge and McChesney, 2018; Lundberg and Abdelzadeh, 2019).

In existing literature, this has been an extensively investigated subject in an attempt to depict a complete model of school environments. We have not only taken into consideration the contributions of Thapa et al. (2013), who identify five dimensions that converge in security, social relations, teaching/learning, institutional environment (both physical and administrative), and process of school improvement; but also the ones from Bradshaw et al. (2014), who suggested that there are three elements that affect the formation of safe and supportive school models, including the variables of commitment, safety, and environment. Both reflect the evolution of research in this area; and, despite their success in the identification of some relevant dimensions of school environment, they still suffer from a lack of variables to consider.

Particularly, as a basis for this study, we reference the contributions of Kutsyuruba et al. (2015) which, as a result from an exhaustive review of published empirical evidence, conclude in a common axis categorization of the school environment named “dimensions of the school climate” that consists of three main categories: (a) physical, refers to the condition of school facilities, the environmental quality of schools, and their relationship with the educational performance and behavior of students; (b) academic, where it is mentioned that the personal skills and characteristics of teachers serve as factors for the development of their students; and finally, (c) social, this specific category suggests that the quality of relationships between members of the school community is fundamental in the configuration of the school climate. These categories shape a conceptual framework that can be regarded as a multidimensional construction of the components and conditions of a positive or safe school environment (Kutsyuruba et al., 2015).

**FIGURE 1:**
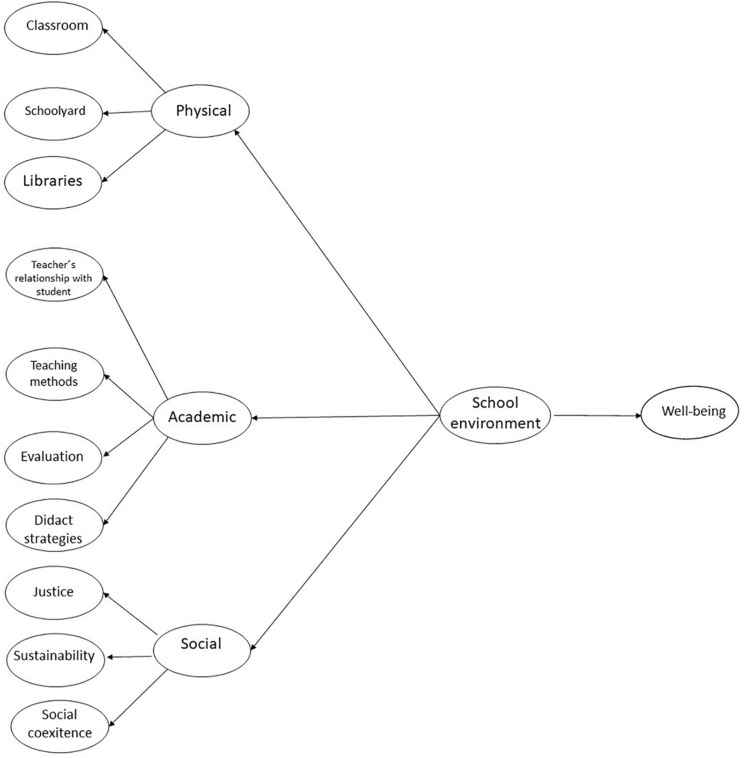
Hypothetic model of the relationship between school environment and well-being.

Our study incorporates and integrates these three dimensions into a variable called school environment and evaluates its impact on student well-being. The participating population consists of children from fifth and sixth grade of primary education in Hermosillo, Mexico. Figure 1 shows the hypothetical model of variable correlations under study, where we propose that the physical dimension comprises the classroom, playground, and library elements; that the academic dimension consists of variables related to students, teaching methodology, didactic strategies, and evaluation; and the social dimension is constituted by justice, sustainability, and social behavior.

## Conceptualization of Categories in the Study

### School Environment

#### Physical

##### Classroom

Space for the delivery of materials that correspond to the areas of basic knowledge where students and teachers interact with furniture that enables individual or group work. Recently it has been mentioned that specific characteristics of the classroom’s physical environment are related to student satisfaction, attitudes, and evaluation of the quality of the course (Fraser, 2015; Han et al., 2019).

##### School yard

Spaces in which students perform educational, civic, recreational, and food-related activities. In a recent study, Dilbil and Basaran (2017) argue that playgrounds positively affected cognitive development and levels of attachment of children to school.

##### Libraries

Space that is well-conditioned to read, learn, and consult a bibliographic collection belonging to the school community where students can interact and work. Schultz-Jones (2011) conducted a study to explain how an evaluation of the learning environment of the school library can be used to demonstrate a positive impact on student performance.

##### Student relationship

In the educational context, the teacher–student relationship is one of the most outstanding academic interactions at the core of the teaching-learning process. Even though this interaction is composed by many other elements, this relationship is the one that plays the most important role when it comes to meeting educational objectives (Bertoglia, 2008). Affective teacher–student communication and interaction plays an important role in building a teacher–student support relationship and a positive classroom environment (Roorda et al., 2011; Poulou, 2014).

##### Teaching methods

The didactic methods are part of the methodological aptitudes that a trainer must have. This means that these types of methods will influence the degree of intervention of the trainer on the student (Calvo, 2006). Teachers’ classroom management practices have a direct impact on the probability of success of their students (Gage et al., 2018). Classroom management and methods are a major challenge for teachers and school administrators, often qualified as the main area of concern for teachers and the most common reason why many choose to leave their profession. Recently, academic research on emotional health, especially during the early years of childhood, has had a greater interest in social and emotional learning and its relationship with the improvement of student behavior (Caldarella et al., 2012).

##### Evaluation

For Bordas and Cabrera (2001), an evaluation system within the classroom will be convenient as long as the students feel like active agents; learn to value their actions and learning, know and understand the curricular objectives; as well as understand the aspects of evaluation in certain tasks. Since the data that teachers receive from their evaluation serve as references for the future, it is necessary to think more deeply about the content of these evaluations, in addition to how we can create conditions for teachers to use this evaluation to inform their instructional methods (Datnow and Hubbard, 2015).

##### Teaching strategies

The term strategy implies reflexive planning to do something by applying any general model used in the classroom (Orlich et al., 2012). Previous studies have concluded that teachers in primary education use different teaching strategies as students gain knowledge through experience, participation in education, express their opinion, and solve problems (Hus and Grmek, 2011).

#### Social

##### Justice

Konow (2003) refers to justice as a virtue that is attached to what is morally correct, concerning the ethics, rationality, natural law, equity, or religion in which they base their foundations.

##### Sustainability

Regarding sustainability, it is important to mention that there are two studies that have prioritized the analysis of sustainable or environmental education. These are “Literature on Environmental Education” (De Castro, 2010) and “Education for Sustainability” by Corral (2010) which required this component to focus more on environmental protection behaviors, forgetting the point that students can obtain various types of benefits when practicing sustainable behaviors (Corral-Verdugo et al., 2015).

##### Social coexistence

Refers to the way students relate with others and how those relationships have important consequences in his/her personal development. Ponferrada-Arteaga and Carrasco-Pons (2010) explain that the emotional expectations that students have about their own school and the degree of recognition and legitimization of the differences manifested by the practices of the school institution influence how students deal with each other at school. A study made by Tian et al. (2016) shows that social support experienced in school is significantly related to subjective well-being.

### Well-Being

Well-being is often interpreted as growth and human satisfaction; it is deeply influenced by the surrounding contexts of people’s lives and, as such, the opportunities for self-realization (Ryff and Singer, 2008). Well-being incorporates the challenges that individuals face in their attempts to fully function and realize their potential (Keyes, 2006; Medina-Calvillo et al., 2013).

One of the reasons why this topic was chosen is because literature that analyzes the conditions of school environments at the basic level requires empiric evidence that proves its impact in children well-being.

## Materials and Methods

The main objective of the study was to test a model where the variable “school environment” is determined by physical, academic, and social dimensions. Our variables were “school environment” and “well-being.” The aim of the study focused on a correlational methodology with the purpose of measuring the degree of relationship between the variables mentioned above (Sampieri et al., 1998). It also has a non-experimental design, since the phenomenon was experienced and measured as it occurred in its natural context. We employed an instrument consisting of different scales that evaluate each of the variables and constructions of the model (Supplementary Data Sheet 1).

### Participants

Four primary schools at the primary level were evaluated, two of them public and two private, all in the city of Hermosillo, Mexico. A total of 405 students were surveyed, 212 females and 193 males, aged between 10 and 12. At the time of the study, the students were in the fifth and sixth grade of primary school.

### Measurements

After deciding on what type of data needed to be collected, the instrument chosen was a survey that consisted of four variables divided in 11 subscales for a total of 63 items. In addition, the survey also included a brief questionnaire inquiring about certain demographic variables related to gender, grade, age, and school.

#### Physical Dimension

This scale assessed the educational spaces such as the classroom, the school yards, and the library. It comprised 15 items and was a semantic differential type scale, where two opposing adjectives are presented and the response is selected from six intermediate values.

#### Academic Dimension

A 24-item scale divided into four subscales: teacher’s relationship with students, teaching methodology, evaluation, and teaching strategies. All subscales were structured with Likert questions, where the response options were “never,” “almost never,” “almost always,” and “always.” In relationship with other students, they were presented with a scale consisting of eight items; the didactic methodology scale has 10 items; the evaluation scale with four items; and, finally, the scale of teaching strategies which includes four items.

#### Social Dimension

Contained three subscales with 11 items, the first one, referring to justice, included four semantic differential type items. The next section, sustainability, was composed of four items also elaborated in Likert scales with four response options going from “never” to “always.” Finally, the social coexistence scale (Fraijo-Sing et al., 2014) evaluated three groups of social interaction, two corresponding to school and one from home, was a Likert scale about satisfaction with five response options ranging from “very unsatisfied” to “very satisfied.”

#### Well-Being

An adaptation for children of the Van Dierendonck (2004) version of Ryff’s (1989) psychological well-being scale (psychological well-being scales, SPWB), from which 13 items were selected, corresponding to the categories of self-acceptance, personal growth, and purpose with life.

Except for the social coexistence and well-being scale, the rest were specifically developed for the purpose of this study and were tested in a regional context (Northern Mexico).

### Procedure

First, a non-random sample was selected; that is, there was a process by which data were extracted to be analyzed, where the universe consists of elementary school students from the city of Hermosillo, Mexico. In the next phase, there was a request for authorization from the directors of the educational institutions to proceed with the application of the instrument. This was carried out in a period of 2 weeks, when students were surveyed in groups in their respective classrooms, without teacher intervention but with their approval.

It is important to emphasize that this instrument was tested as reliable and valid by comparing the magnitude of the different variables and indicators. Once the surveys were answered and the numerical valuations of variables were made, we obtained ranges of values for the responses, as well as the different trends obtained. Through this data analysis, we transformed the data into information that was used to answer our research questions by using the Statistical Package for Social Sciences (SPSS v21.0). Using this, we analyzed the psychometric properties and construct validity through exploratory factor analysis, reliability through Cronbach’s alpha, analysis of descriptive data of each of the scales, and correlation coefficients between the scales (Supplementary Table 1).

Subsequently, we tested the structural model using the statistical program EQS. First, we analyzed the measurement models of each of the variables. Then, we performed a structural model analysis to test the model of school environments using procedures in first instance plot development (sets of two variables). Likewise, first and second order variables were formed.

## Results

Table 1 shows the correlation matrix of the measured variables of scholar environment and their internal consistencies. The Cronbach’s alpha values in all used scales turned out to be appropriate, indicating an acceptable reliability coefficient of the instruments. Overall, the correlations go from moderate, but statistically significant, to strongly correlated.

**TABLE 1:** Univariate statistics and their relationship to school environment and well-being.

	$\bar{X}$	σ	Alpha	PH	AC	SO	WB
PH	3.7	0.66	0.79	1			
AC	3.1	0.44	0.88	0.407**	1		
SO	3.5	0.60	0.74	0.606**	0.647**	1	
WB	1.9	0.67	0.67	0.342**	0.284**	0.344**	1

*n* = 411; $\bar{X}$ = mean; σ = standard deviation; PH = physical dimension; AC = academic dimension; SO = social dimension; WB = well-being. Pearson *p < 0.05. **p < 0.001.

### Structural Model

**FIGURE 2:**
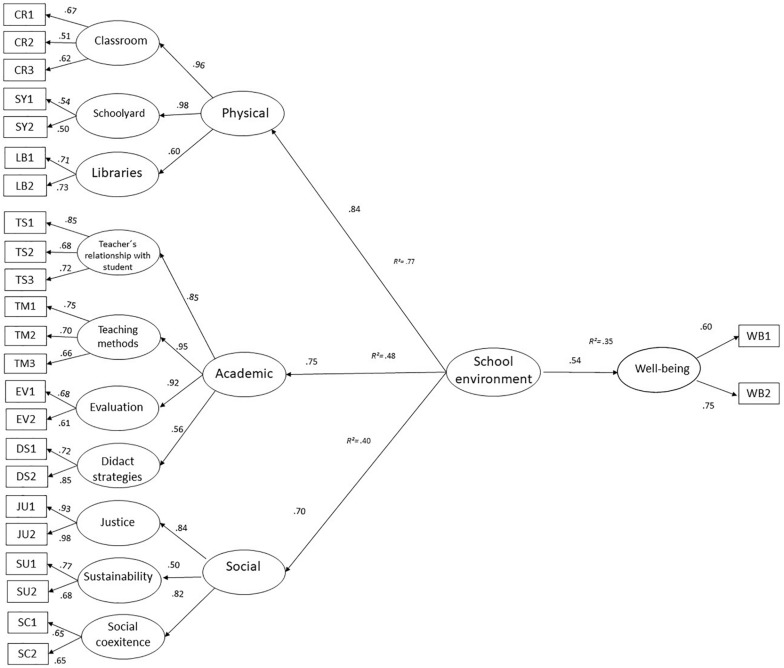
Structural model of the relationship between school environment and well-being. Goodness of fit: *X^2^* = 570.99 (307 *df*), *p* = 0.000, *BBNFI* = 0.90, *BBNNFI* = 0.91, *CFI* = 0.93, *RMSEA* = 0.04. Well-being *R^2^* = 0.35.

Figure 2 shows the structural model that illustrates the relationship between the variables “school environment” (composed of physical, academic, and social factors) and “well-being.” In reference to model fitting and its interpretation, researchers use numerous goodness-of-fit indicators to assess a model. Some common fit indexes are the normed fit index (NFI), non-normed fit index (NNFI), and comparative fit index (CFI) (Hu and Bentler, 1999). Absolute fit indexes were also employed to evaluate the degree to which the model proposed and how the actual data variance–covariance matrices compare. Some absolute fit indexes include the chi-square statistic and the standardized root-mean-square residual (Bentler, 1995). We can verify that the indicators of goodness of statistical adjustment (X^2^ = 570.99, 307 df, *p* = 0.000) were not significant, so there are no apparent reasons, in mathematical matter (Corral-Verdugo, 1995), to discard this model and the relationships that are illustrated in it. On the other hand, it should also be noted that the goodness of fit indexes adjustments (BBNFI = 0.90, BBNNFI = 0.91, CFI = 0.93, RMSEA = 0.04.) show that the structural model is supported by the amount of data that was presented in this sample, since all values are equal to or greater than 0.90 (Bentler, 1990).

## Discussion

Our research was presented with the chance to provide additional empirical evidence to the conclusions of the work of Kutsyuruba et al. (2015), who determined integrative categories associated with studies on school climate and proposed a three-dimensional model: physical, academic, and social. Other studies have offered a conceptual framework derived from a multidimensional construction of components and conditions of a positive school environment (Wang and Degol, 2016). In addition to confirming the relevance of this theoretical–conceptual approach, we recognized a causal relationship between the school environment and the well-being of elementary education students who participated in the study (Aldridge and McChesney, 2018).

The hypothetical model that guided this research was confirmed by the structural model’s second order factor called “school environment” which was shaped by the three dimensions suggested by Kutsyuruba et al. (2015): physical, academic, and social. In turn, the “school environment” had an effect on the “well-being” variable (Ryff and Singer, 2008), which also allowed us to verify the relevance of the suggestions made by Corral-Verdugo et al. (2015) in their review and conceptualization of a “positive school.”

Hypothesized first-order factors were also conformed by their respective measures and by the nesting of their variables. Confirming these relationships leads us to conclude that the present estimation and evaluation of the school environment dimension model was measured in a valid and pertinent manner for this construct. Results obtained by this model support the ideas of the three-dimensional construct of Kutsyuruba et al. (2015) and confirm this theoretical model in the reality of children of fifth and sixth grade of basic education in Hermosillo, Mexico.

Such remarks allow for some reassurance that we have established some of the variables that could influence a positive school climate (Bosworth et al., 2011; Aldridge and McChesney, 2018). In the three dimensions proposed by the model, we can also identify the actions required in order to impact on well-being and its relationship with the academic achievement of the students (Maxwell et al., 2017), their ways of relating to teachers (Roorda et al., 2011), and the relationships they establish with peers and others in their environment (Tian et al., 2016).

In other regards, this work suffers from limitations notably related to methodological aspects and the means used to collect data. Even when speaking about the validity of the instruments and statistical procedures that account for their reliability, the surveys used for this analysis were specifically developed for the purpose of this study on a non-random sample, which may compromise the generalizability of our findings, despite obtaining acceptable goodness of fit indexes. Therefore, we recommend future research should therefore seek to address this issue by devising a specific method for gathering data on random samples by the means of surveys.

A key strength of this research lies within the integration of the three aspects considered in our model. Some studies have discussed variables related to well-being. For instance, how the physical design of space affects learning and the well-being of children (Martin, 2016); how teacher support and the ways it is perceived by students impacts well-being (Reddy et al., 2003); and also, the way social relationships with companions and peers may serve as a protective factor for well-being (Lindberg and Swanberg, 2006). However, gathering all of these variables into a single model can be considered to be a significant step forward in the study of student well-being, as well as which variables should be considered in order to design and promote the implementation of programs concerning well-being in school environments.

## Conclusion

The posture of a school environment factor constituted by physical, social, and academic components was verified and adequately supported by the data gathered in our study and the structural model obtained in Figure 2. The school environment factor also correlated significantly with a measure of well-being as proposed by our hypothetic model. Moreover, our measure of school environment was found to be a valid one given regarding internal consistency where all factors have a reasonable level of reliability; we can see that all the variables show acceptable correlation values as we also consider the goodness of fit indexes obtained.

Our model confirmed that, in order to promote subjective well-being, schools must facilitate the optimal development of people by accepting that all students possess differentiated strengths, recognize its students’ abilities, and offer school environments that imply positivity in aspects concerning the physical, social, and didactic spheres of school life. Insights into these aspects are expected to contribute to a better understanding of how they correspond harmoniously with the abilities and expectations of the students (Corral-Verdugo et al., 2015; Maxwell et al., 2017). The potential implementation of these findings has been widely described in literature. A school should aim its goals toward the promotion of the subjective well-being of its students, without neglecting the purposes of developing academic and cognitive skills (Huebner et al., 2009).

In order to design an accurate system, knowledge of the factors that contribute to well-being in school environments is necessary. The application of these research findings should be focused on the advocacy of curricula that embodies these factors, in such a manner that may comprise better practices in school environments (Bird and Markle, 2012). A more interesting and practical scenario would be if findings such as the ones found in this study could be oriented toward the outlining or amelioration of public education programs dedicated to student’s prosperity, learning, and well-being.

## Data Availability Statement

The datasets generated for this study are available on request to the corresponding author.

## Ethics Statement

The studies involving human participants were reviewed and approved by Comité de ética en Investigación de la Universidad de Sonora. Written informed consent to participate in this study was provided by the participants’ legal guardian/next of kin.

## Author Contributions

CT-F and BF-S contributed by writing, reviewing, and editing. CT-F and VC-V contributed with conceptualization and design of this study. GG-T ran formal analysis and organized databases. CT-F contributed by supervising this study and its methodological tasks (methodology) were designed by CT-F and BF-S. GG-T and MM-B provided the writing of the original draft. All authors contributed to manuscript revision and read and approved the submitted version.

## Conflict of Interest

The authors declare that the research was conducted in the absence of any commercial or financial relationships that could be construed as a potential conflict of interest.
