# Supplementary material for: School Environments and Elementary School Children’s Well-Being in Northwestern Mexico
Source: Front Psychol. 2020 Mar 19;11:510. doi: 10.3389/fpsyg.2020.00510 (PMC7098376; doi:10.3389/fpsyg.2020.00510)
Supplement: Supplementary file 1 [file Table_1.docx]

Supplementary Material

| School Environment | | | | | | | | | | |
| --- | --- | --- | --- | --- | --- | --- | --- | --- | --- | --- |
| Items | Factors and factor loadings. | | | | | | | | | |
|  | Classroom  .78 | Schoolyard  .71 | Libraries  .47 | Justice  .84 | Sust.  .72 | Social Co.  .67 | Teacher relation  .87 | Methodology  .88 | Eval.  .90 | Didactic  .65 |
| AU1 | .55 |  |  |  |  |  |  |  |  |  |
| AU2 | .44 |  |  |  |  |  |  |  |  |  |
| AU3 | .46 |  |  |  |  |  |  |  |  |  |
| AU4 | .44 |  |  |  |  |  |  |  |  |  |
| AU5 | .30 |  |  |  |  |  |  |  |  |  |
| AU6 | .58 |  |  |  |  |  |  |  |  |  |
| AU7 | .41 |  |  |  |  |  |  |  |  |  |
| PA1 |  | .60 |  |  |  |  |  |  |  |  |
| PA2 |  | .31 |  |  |  |  |  |  |  |  |
| PA3 |  | .52 |  |  |  |  |  |  |  |  |
| PA4 |  | .49 |  |  |  |  |  |  |  |  |
| BI1 |  |  | .52 |  |  |  |  |  |  |  |
| BI2 |  |  | .62 |  |  |  |  |  |  |  |
| BI3 |  |  | .57 |  |  |  |  |  |  |  |
| BI4 |  |  | .78 |  |  |  |  |  |  |  |
| JU1 |  |  |  | .61 |  |  |  |  |  |  |
| JU2 |  |  |  | .57 |  |  |  |  |  |  |
| JU3 |  |  |  | .64 |  |  |  |  |  |  |
| JU4 |  |  |  | .58 |  |  |  |  |  |  |
| SU1 |  |  |  |  | .61 |  |  |  |  |  |
| SU2 |  |  |  |  | .54 |  |  |  |  |  |
| SU3 |  |  |  |  | .64 |  |  |  |  |  |
| SU4 |  |  |  |  | .58 |  |  |  |  |  |
| CS1 |  |  |  |  |  | .75 |  |  |  |  |
| CS2 |  |  |  |  |  | .62 |  |  |  |  |
| CS3 |  |  |  |  |  | .22 |  |  |  |  |
| RE1 |  |  |  |  |  |  | .73 |  |  |  |
| RE2 |  |  |  |  |  |  | .60 |  |  |  |
| RE3 |  |  |  |  |  |  | .57 |  |  |  |
| RE4 |  |  |  |  |  |  | .54 |  |  |  |
| RE5 |  |  |  |  |  |  | .60 |  |  |  |
| RE6 |  |  |  |  |  |  | .51 |  |  |  |
| RE7 |  |  |  |  |  |  | .70 |  |  |  |
| ME1 |  |  |  |  |  |  |  | .57 |  |  |
| ME2 |  |  |  |  |  |  |  | .59 |  |  |
| ME3 |  |  |  |  |  |  |  | .47 |  |  |
| ME4 |  |  |  |  |  |  |  | .59 |  |  |
| ME5 |  |  |  |  |  |  |  | .50 |  |  |
| ME6 |  |  |  |  |  |  |  | .45 |  |  |
| ME7 |  |  |  |  |  |  |  | .35 |  |  |
| ME8 |  |  |  |  |  |  |  | .53 |  |  |
| ME9 |  |  |  |  |  |  |  | .49 |  |  |
| EV1 |  |  |  |  |  |  |  |  | .53 |  |
| EV2 |  |  |  |  |  |  |  |  | .61 |  |
| EV3 |  |  |  |  |  |  |  |  | .56 |  |
| EV4 |  |  |  |  |  |  |  |  | .47 |  |
| FR1 |  |  |  |  |  |  |  |  |  | .60 |
| FR2 |  |  |  |  |  |  |  |  |  | .72 |
| FR3 |  |  |  |  |  |  |  |  |  | .59 |
| FR4 |  |  |  |  |  |  |  |  |  | .71 |

**Table 1.** Confirmatory factor analysis and factor loadings of school environment scale. All factor loads are significant (p <0.05). Goodness of fit indices: X² = 1543.63 (1123 gl.), P = .000, , BBNFI = .90, BBNNFI = .90, CFI = .91, RMSEA = .03.
