# Supplementary material for: School Environments and Elementary School Children’s Well-Being in Northwestern Mexico
Source: Front Psychol. 2020 Mar 19;11:510. doi: 10.3389/fpsyg.2020.00510 (PMC7098376; doi:10.3389/fpsyg.2020.00510)
Supplement: Supplementary file 2 [file Data_Sheet_1.docx]

Supplementary Material

INFORMED ASSENT

**School environments and its relationship with well-being in elementary school children in northwestern Mexico.**

The purpose of the present research study is to explain the relation between elementary school environment and well-being in children. An instrument that includes questions with various response options will be applied to scholar children-

Hello! My name is _____________________________ and I work in the Psychology Department at the University of Sonora. Currently, a study is being conducted to know more about the school environment and the well-being presented by children in your school, and we want to ask for your support.

Your participation in the study consists in answering a few questions.

Your participation in the study is voluntary, that means, even when your parents said that you can participate, if you do not want to do it, you can say no. It is your decision whether you participate in the study or not. Also, it is important for you to know that, if you do not want to continue in the study at any moment or if you do not want to answer a question, there will no problem.

All the information you provide to us/the measurements we will obtain, will help us to explain how you feel in your school.

This information will be confidential. It means that we are not going to tell nobody your answers (OR MEASUREMENT RESULTS), only the people who are part of the research team.

If you agree to participate, please mark with a check mark **(✓)** the box below and write your name.

If you don’t agree to participate, do not put a check mark **(✓)** and don’t write your name.

Yes, I agree to participate.

Name: __________________________________________

Name and signature of the person who obtains the assent:

______________________________________________________________

Date: _____________________.

The questions in this questionnaire are about your life as a student in your school; I would like you to answer the questions as honest as you can, because your answers will be very useful for the research study.

Please remember to read each question carefully before you answer, there is no right or wrong answer. Your opinion is very important to this research study. The information you provide it is confidential.

**How old are you?** _______ **which** **grade are you in?** _________ **Gender:**  M F

**School Environment**

Physical Dimension

**Instruction**: Read carefully each question about the conditions of your school and mark a single number in the 0 – 5 scale.

***Classroom***

**1.- Classroom furniture is in good condition and clean?**

*Very bad and dirty* 0 1 2 3 4 5 *Good condition and clean*

**2.- The temperature inside the classroom is**

*Unpleasant* 0 1 2 3 4 5 *Pleasant*

**3.- I value the natural light (windows) in the classroom as**

*Limited* 0 1 2 3 4 5 *Adequate*

**4.- The ventilation inside the classroom is**

*Insufficient* 0 1 2 3 4 5 *Very appropiate*

**5.- I consider the artificial lighting (spotlights, lamps) in the classroom as**

*Limited* 0 1 2 3 4 5 *Adequate*

**6.- The size of the classroom and the distribution of furniture and equipment are**

*Nothing appropriate* 0 1 2 3 4 5 *Very appropiate*

**7.- The acoustic inside the classroom is**

*Affected by outside noise* 0 1 2 3 4 5 *Outside noise free*

**Instruction**: Read carefully each question about the school yard and the library of your school and mark a single box with an “**X**”.

***The school yard are:***

| 1. Dirty | **0** | **1** | **2** | **3** | **4** | **5** | Clean |
| --- | --- | --- | --- | --- | --- | --- | --- |
| 2. Reduced   space | **0** | **1** | **2** | **3** | **4** | **5** | Enough space |
| 3. Insecure | **0** | **1** | **2** | **3** | **4** | **5** | Safety |
| 4. Not enough  equipped for  games | **0** | **1** | **2** | **3** | **4** | **5** | Enough equipped for games |

***About the school library I think it is:***

| 1. Thin light | **0** | **1** | **2** | **3** | **4** | **5** | Strong light |
| --- | --- | --- | --- | --- | --- | --- | --- |
| 2. A few furniture | **0** | **1** | **2** | **3** | **4** | **5** | A lot furniture |
| 3. Not enough  options of books | **0** | **1** | **2** | **3** | **4** | **5** | Wide variety of books |
| 4. Small space | **0** | **1** | **2** | **3** | **4** | **5** | Enough space |

Academic Dimension

**Instruction**: Read carefully each question about the relationship between students and teachers of your school and mark a single answer between “never” to “always”.

***Relationship/interaction with students***

1. **My teacher is accessible and willing to help us (The teacher wants to help us).**

Never Almost never Almost always Always

1. **If we ask any questions in class, my teacher questions us and guides us.**

Never Almost never Almost always Always

1. **It’s easy talk with my teacher even when it’s not class time.**

Never Almost never Almost always Always

1. **My teacher is interested in what we do on weekends, sports, fun, hobbies, journeys or other activities.**

Never Almost never Almost always Always

**5. My teacher answers everyone’s questions.**

Never Almost never Almost always Always

**6. My teacher is interested in what we do outside of school.**

Never Almost never Almost always Always

**7. My teacher treat us well, he’s/she’s kind.**

Never Almost never Almost always Always

**Instruction**: Read carefully each question about the activities your teacher does and mark a single answer between “never” to “always”.

***Teaching Methodology***

1. **My teacher concerns if a student doesn’t understand something we check in class.**

Never Almost never Almost always Always

1. **My teacher speaks in a way that we understand.**

Never Almost never Almost always Always

1. **My teacher explains the importance of learning what we have seen in class and how we can apply it in our lives.**

Never Almost never Almost always Always

1. **My teacher motivates us to participate during class.**

Never Almost never Almost always Always

1. **My teacher uses examples, concept maps, graphics or images to support what he/she explains in class.**

Never Almost never Almost always Always

1. **My teachers send reports to our parents about our progress in class.**

Never Almost never Almost always Always

1. **My teacher uses pictures, movies and videos to support the topics seen in class.**

Never Almost never Almost always Always

1. **My teacher leaves Project development to support topics seen in class.**

Never Almost never Almost always Always

1. **My teacher uses support books different to the textbooks.**

Never Almost never Almost always Always

***Evaluation***

1. **I know how my teacher will evaluate each subject at the beginning of each topic**

Never Almost never Almost always Always

1. **We are clear about what my teacher ask us to do in every class.**

Never Almost never Almost always Always

1. **When my teacher gives us the grades, he/she explains to us why we get that grade and can check it if we think that might be an error.**

Never Almost never Almost always Always

1. **My teacher returns class assignments pointing out how to correct our mistakes.**

Never Almost never Almost always Always

***Teaching strategies***

***How often your teacher in class:***

1. **Create experiments to explain a natural phenomenon.**

Never Almost never Almost always Always

1. **Prepare together with the class group proposals for the care of the environment or nature.**

Never Almost never Almost always Always

1. **Prepare mathematical exercises with examples of the environment or nature.**

Never Almost never Almost always Always

1. **Leave us reading material with topics about environmental issues: nature, how to take care of plants and animals, etc.**

Never Almost never Almost always Always

Social Dimension

**Instruction**: Read carefully each question about the rules of your school and mark a single number in the 0 – 5 scale.

***Justice***

**1.- The school rules are applied in my classroom:**

Nothing equal 0 1 2 3 4 5 Very equal

**2.- The homework and exam evaluation system is:**

Not equally applied 0 1 2 3 4 5 Equally applied

**3.- Sanctions and rewards:**

Not accomplished 0 1 2 3 4 5 Totally accomplished

**4.- My participation in important decision making:**

Not considered 0 1 2 3 4 5 Very considerate

**Instruction**: Read carefully each question about the environmental behavior of your school and mark a single answer between “never” to “always”.

***Sustainability***

1. **The reuse of educational materials and garbage recycling is promoted in my school.**

Never Almost never Almost always Always

1. **School environmental care campaigns: saving electricity, water care, reforestation is an important
    part of school programs.**

Never Almost never Almost always Always

1. **Healthy school life is promoted through festivals, sports games, competitions or special
    presentations as talent show, for example.**

Never Almost never Almost always Always

1. **There are activities in the school program that promote respectful communication between students and
    teachers, such as plays, plenary session, special training, sign campaigns or reflection events
    (communication courses-workshops).**

Never Almost never Almost always Always

**Social coexistence**

Mark below the level of satisfaction it generates the relation you live in these different groups, **mark your answer with an “X”.**

| **Social group** | **Very unsatisfied** | **Unsatisfied** | **Neutral** | **Satisfied** | **Very satisfied** |
| --- | --- | --- | --- | --- | --- |
| My classroom | **0** | **1** | **2** | **3** | **4** |
| My school | **0** | **1** | **2** | **3** | **4** |
| My house | **0** | **1** | **2** | **3** | **4** |

**Well-being**

**Instruction**: Read carefully each question and mark a single answer between “everyday” to “never”.

1. **I feel confident and positive about myself.**

Everyday Almost everyday Sometimes Almost never Never

1. **I feel that my friends help me in so much things.**

Everyday Almost everyday Sometimes Almost never Never

1. **I feel good when I think about what I’ve done in the past.**

Everyday Almost everyday Sometimes Almost never Never

1. **I feel good when I think about what I will do in the future.**

Everyday Almost everyday Sometimes Almost never Never

1. **My purpose in life makes me feel good.**

Everyday Almost everyday Sometimes Almost never Never

1. **I like most aspects of my personal characteristics .**

Everyday Almost everyday Sometimes Almost never Never

1. **I am not afraid to voice my opinions, even when they are in opposition to the opinions of most people.**

Everyday Almost everyday Sometimes Almost never Never

1. **I have a sense of direction and purpose in life.**

Everyday Almost everyday Sometimes Almost never Never

1. **With time, I feel that I keep learning about myself.**

Everyday Almost everyday Sometimes Almost never Never

1. **I feel proud about who I am and the life I’m taking.**

Everyday Almost everyday Sometimes Almost never Never

1. **I know that I can trust my friends, and they know they can trust me.**

Everyday Almost everyday Sometimes Almost never Never

1. **I have the sense that I have developed a lot as a person over time.**

Everyday Almost everyday Sometimes Almost never Never

1. **For me, life has been a continuous process of learning, changing, and growth.**

Everyday Almost everyday Sometimes Almost never Never
